# Operational challenges in managing Isoniazid Preventive Therapy in child contacts: A high-burden setting perspective

**DOI:** 10.1186/1471-2458-11-544

**Published:** 2011-07-08

**Authors:** Susan S van Wyk, Anthony J Reid, Anna M Mandalakas, Donald A Enarson, Nulda Beyers, Julie Morrison, Anneke C Hesseling

**Affiliations:** 1 Desmond Tutu TB Centre, Department of Paediatrics and Child Health, Stellenbosch University, Cape Town, South Africa; 2 Department of Pediatrics, School of Medicine, Case Western Reserve University, Cleveland, Ohio, USA; 3 Medecins Sans Frontieres, Operational Centre Brussels, Belgium; 4 International Union Against Tuberculosis and Lung Disease, Paris, France

## Abstract

**Background:**

The study was conducted at a high TB-HIV burden primary health community clinic in Cape Town, South Africa. We describe the management of children under five years of age in household contact with a smear and/or culture-positive adult TB case.

**Methods:**

This study was a record review of routinely-collected programme data.

**Results:**

A total of 1094 adult TB case folders were reviewed. From all identified contacts, 149 children should have received IPT based on local guidelines; in only 2/149 IPT was initiated. Management of child contacts of sputum smear and/or culture-positive compared to sputum-negative TB patients were similar.

**Conclusions:**

IPT delivery to children remains an operational challenge, especially in high TB-HIV burden communities. A tool to improve IPT management and targeting sputum smear and/or culture-positive TB child contacts may overcome some of these challenges and should be developed and piloted in such settings.

## Background

South Africa has an estimated tuberculosis (TB) incidence rate ranking first in the world and has one of the highest reported childhood TB rates [1,2]. The estimated incidence of childhood TB in the Western Cape Province of South Africa was 620/100 000/year in 2007 [unpublished data, Western Cape Department of Health].

The risk of developing TB in children can be substantially reduced by administration of Isoniazid Preventive Therapy (IPT) in those infected with *Mycobacterium tuberculosis (M.tb)*[3,4]. Children in high TB-burden settings are frequently exposed to cases with infectious TB at home, leading to infection with *M.tb *at a young age. Furthermore, the risk of developing TB is the highest in children under five years of age [5-7]. Based on the high risk of disease progression and efficacy of post-exposure prophylaxis, IPT is therefore recommended by the World Health Organization (WHO) and South Africa National TB Programme (SANTP) for all children under five years of age and all HIV infected children, regardless of age, in contact with an infectious (smear and/or culture-positive) TB case [8,9].

Despite these recommendations being in place for more than 20 years, implementation of IPT in children appears to be suboptimal in South Africa, where four studies on IPT delivery in children have been published to date [10-13]. One hospital-based study from Cape Town, showed that 117/182 (64.3%) of children admitted with culture-confirmed TB had missed opportunities for IPT [10]. Three community-based studies were conducted at two clinics with annual TB caseloads of 159 and 156 respectively in 2007 [unpublished data, Cape Town City Health, 2008]; 40% of TB cases received HIV testing of whom 15% were HIV-positive at these clinics [14]. These studies showed that fewer than 17% of eligible children 0-5 years initiated IPT and fewer than 15% who initiated IPT completed 4 months' therapy [11,13].

Given the increased emphasis on IPT delivery to vulnerable populations including children and HIV-infected individuals we aimed to investigate health system challenges in IPT delivery to children in a large TB clinic with high TB and HIV case loads [15]. The primary objective was to describe the routine management of children younger than five years of age in household contact with a sputum smear and/or culture-positive adult TB case at a high-burden primary health community clinic. A secondary objective was to assess whether adult sputum smear status was a determinant for IPT delivery in child contacts.

## Methods

### Design

This study was a record review of routinely-collected programme data.

### Setting

The study was conducted at a clinic in Khayelitsha, Cape Town, South Africa, an urban and densely populated community with a current estimated population of 406 779 [16]. In Khayelitsha 80% of the population live in informal dwellings, 86% have not completed high school, 47% are unemployed and 55% of households are judged to be below the poverty line [17]. The TB caseload at the study clinic was almost 1000 (33% new smear-positive) patients per annum with a cure rate of 80.5% in new smear-positive cases in 2007 [unpublished data, Cape Town City Health, 2008]; 90% of all TB cases routinely received HIV testing of whom 70% were HIV-positive [18].

Active contact tracing through home visits by clinic personnel is not routinely recommended by the SANTP; rather, guidelines recommend that children under five years of age who are in close (household) contact with an adult TB case, should be identified, invited to the clinic for TB screening and initiate IPT once active TB disease is excluded. Children in contact with multi-drug resistant (MDR) TB cases are referred to two off-site specialized clinics for MDR screening and prophylaxis.

### Sample

This study included, as entry point, all adults who initiated TB treatment during 2008 according to the electronic TB treatment register (compiled at district level from paper based TB treatment registers), and all children younger than five years of age documented to be in contact with these adult TB cases.

### Data collection and outcome measures

An adult TB case was defined as any patient older than 15 years of age who was initiated on anti-tuberculosis treatment during the defined period. An "infectious" case was defined as a TB case with either a sputum smear or culture-positive result. Patients with concurrent pulmonary and extra-pulmonary disease were defined as pulmonary. Eligible children were defined as all children under five years of age reported to be living in the same household as a sputum smear and/or culture-positive adult TB case and therefore eligible for IPT, as per national guidelines. TB screening of the child contact was defined as any documentation of evaluation for TB, including clinical evaluation, tuberculin skin testing, chest radiography and tests of sputum or gastric washing. A patient folder was defined as absent if it could not be retrieved from the clinic after two consecutive days of searching.

Electronic TB registers were used to identify all adult TB cases diagnosed from January through December 2008. Using a previously used data extraction tool [13], individual TB folders of these adult TB cases were manually reviewed and contact data extracted. Contact data of eligible children were then used to identify potential individual child clinic folders. Using the data extraction tool [13], information on TB screening and IPT initiation and adherence were extracted from the child clinic folders. Due to the different system supporting management of children in contact with drug-resistant TB cases, these children were excluded from our study.

### Ethics

The protocol was approved by the Ethics Advisory Group of The Union and the Committee for Human Research of Stellenbosch University. Cape Town City Health Directorate gave permission for the study.

## Results

From the TB register, 1255 adult TB cases were identified during the study period. Among these, 1094 individual adult TB case folders could be retrieved. Demographic details of adult TB cases are shown in Table 1.

**Table 1:** Demographic data of adult TB cases identified from the electronic clinic TB treatment register

	Adult TB cases						
	Total with individual TB folder	Sputum smear positive	Sputum smear negative, culture positive	Sputum smear negative and culture negative	Sputum status unknown	Extra-pulmonary	No folder identified
	1094	525 (48%)	158 (14%)	152 (14%)	113 (10%)	146 (13%)	160
**Age (years) **Med (IQR)	32 (26-41)	31 (25-40)	35 (29-43)	32 (26-41)	34 (28-42)	33 (27-41)	32.5 (28-40)
**Male (%)**	565 (52)	303 (58)	69 (44)	64 (42)	57 (50)	72 (49)	85 (53)
**HIV-infected (%)**	503 (46)	201 (38)	83 (53)	82 (54)	54 (48)	83 (57)	69 (43)
**HIV unknown (%)**	353 (32)	172 (33)	49 (31)	54 (36)	40 (35)	38 (26)	70 (44)
**MDR (%)**	3	3	0	0	0	0	13 (8)
**Retreatment (%)**	296 (27)	135 (26)	62 (39)	38 (25)	34 (30)	27 (18)	58 (36)

From folders that were available for review, a total of 683 (62%) [525 smear-positive and 158 culture-positive] adults were identified as sputum smear and/or culture-positive ("infectious") and 411 (38%) as sputum-negative/unknown or extra-pulmonary ("non-infectious"). Three smear-positive adults were MDR TB cases and therefore excluded from the analyses.

**Figure 1:**
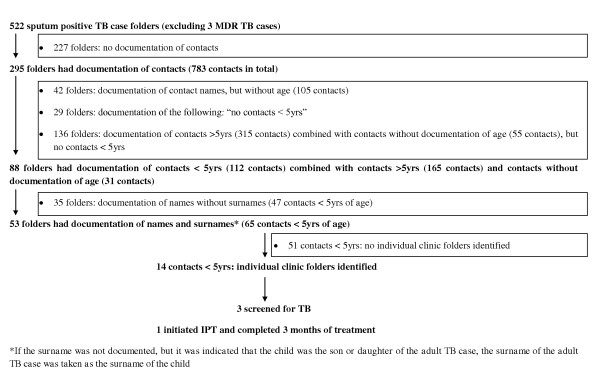
**Details of contact identification and management for sputum smear-positive TB cases**.

**Figure 2:**
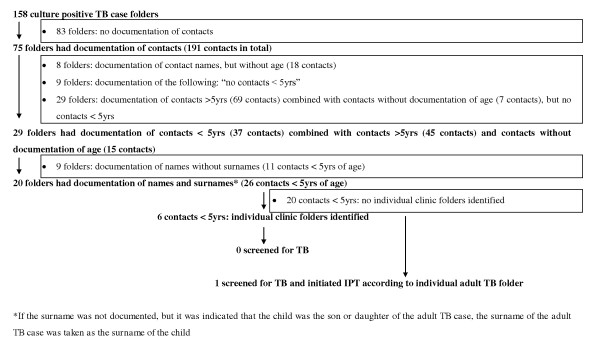
**Details of contact identification and management for sputum smear-negative, culture positive TB cases**.

One hundred and forty nine (15%) of all 974 documented contacts of sputum smear and/or culture-positive adult TB cases could be identified as being under five years of age. Only four of these eligible children were screened for TB, two were initiated on IPT and one child had documentation of continuation of IPT and completed three months of treatment according to the clinic records. Details of contact identification and management for sputum smear and/or culture-positive TB cases are provided in figures 1 and 2.

Fifty six (15%) of the 379 documented contacts of sputum-negative/unknown or extra-pulmonary adult TB cases could be identified as being under five years of age. Two of these children who were not eligible for IPT as per guidelines, were screened for TB and one was initiated on IPT. None had documentation of continuation or completion of IPT.

**Figure 3:**
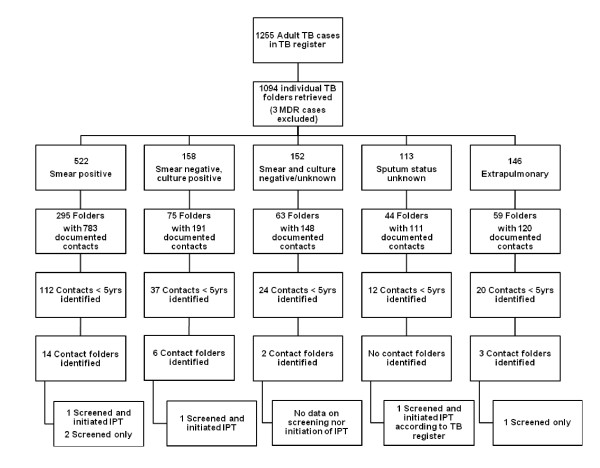
**Data sources identified for adult TB cases and their identified child contacts in conjunction with documentation of TB screening and IPT delivery**.

Although formal tests of significance were not completed due to the small number of children initiated on IPT, the identification of contacts and documentation of IPT appeared to be similar for sputum and/or culture-positive and sputum-negative adult TB cases (Figure 3). This documentation was also similar for child contacts of both HIV-positive and negative adults.

## Discussion

We report on a large study of programmatic IPT management in children at a clinic with a high TB and HIV burden in South Africa. Only 1% (2/149) of children who could be identified as "eligible" for IPT had any routine documentation of IPT initiation. Children in contact with sputum smear and/or culture-positive adults were not prioritized for prophylaxis, as recommended by WHO [8]. Routine recording of contact identification and management was neither systematic nor consistent. At each step along the cascade of IPT delivery- identification of infectious adult TB cases, identification of high-risk child contacts, screening for TB and initiation of IPT - children were missed.

The results of our study revealed considerably worse IPT delivery to children compared with previous data from South Africa and other settings [10-13]. In one South African study, where the same methods were used, 17% (4/24) of eligible children had documentation of initiation of IPT [13]. Similarly, in other settings, data on IPT delivery to children showed IPT initiation rates of 8% in Malawi [19,20] and 19% in India [21].

Reasons for this poor observed performance have not yet been fully explored. However, a higher TB clinic caseload and a higher prevalence of TB-HIV co-infection, with the concomitantly increased workload on TB staff, in this study may be a reason for the extremely poor performance. A simple clinic-based management tool, such as an IPT register [8], which is linked to the adult TB register data, may help personnel to prioritise IPT care for children in contact with sputum smear and/or culture-positive adults, while spending less time on children in contact with sputum-negative adults. Another reason for poor performance may be insufficient knowledge of contact management amongst health workers. In a recent Indian study, focus group discussions among health care workers suggested that poor provision of documentation and a lack of detailed knowledge about required procedures may be reasons for poor IPT delivery [21]. Finally, in the face of heavy demands for care of TB-HIV co-infection, healthcare workers may not consider IPT particularly important. Thus, we recommend that knowledge and attitudes amongst healthcare personnel and parents regarding IPT delivery should also be investigated.

There are some limitations in this study. We could only report on routinely-documented data and this may differ from actual IPT delivery. In addition, it was carried out at a single site and the results may not be applicable to other similar contexts. Finally, we do not report on HIV-infected child contacts older than five years of age.

## Conclusions

This study suggests that IPT delivery to children remains an operational challenge, especially in settings with a high burden of TB and HIV. A tool to improve IPT management and targeting sputum smear and/or culture-positive TB child contacts may overcome some of these challenges. Education regarding the techniques and importance of IPT may also improve performance.

## Competing interests

The authors declare that they have no competing interests.

## Authors' contributions

SSVW conceived of the study, participated in the design, was responsible for data acquisition, participated in data analysis and interpretation and drafted the manuscript. ACH and AJR conceived of the study, participated in the design and drafted the manuscript. JM participated in data acquisition. AMM, DAE and NB revised the article critically for intellectual content. All authors read and approved the final manuscript.

## Pre-publication history

The pre-publication history for this paper can be accessed here:

http://www.biomedcentral.com/1471-2458/11/544/prepub
